# Accurate Measurements of the Zero-Dispersion Wavelength in Optical Fibers

**DOI:** 10.6028/jres.102.023

**Published:** 1997

**Authors:** S. E. Mechels, J. B. Schlager, D. L. Franzen

**Affiliations:** National Institute of Standards and Technology, Boulder, CO 80303-3328

**Keywords:** chromatic dispersion, dispersion slope, nonlinear four-wave mixing, optical fiber, optical fiber bandwidth, polarization-mode dispersion, Standard Reference Material, telecommunication systems, zero-dispersion wavelength

## Abstract

We have developed a frequency-domain phase shift system for measuring the zero-dispersion wavelength and the dispersion slope of single-mode optical fibers. A differential phase shift method and nonlinear four-wave mixing technique were also investigated. The frequency-domain phase shift method is used to produce Standard Reference Materials that have their zero-dispersion wavelengths characterized with an expanded uncertainty (*k* = 2) of ± 0.060 nm.

## 1. Introduction

This paper reports the development of a Standard Reference Material (SRM) which characterizes the zero-dispersion wavelength (*λ*_0_) and the dispersion slope (*S*_0_) at *λ*_0_ of single-mode optical fibers. We have documented a system which measures the dispersion of both dispersion-unshifted (*λ*_0_ near 1.3 μm) and dispersion-shifted fibers (*λ*_0_ near 1.55 μm). While the principal system uses the frequency-domain phase shift technique [1], differential phase shift [2] and four-wave mixing techniques [3] have also been investigated. The fiber SRMs have their *λ*_0_ value measured with a combined expanded uncertainty (coverage factor *k* = 2, thus a 2 standard deviation estimate) of 0 ± 0.060 nm. Dispersion slope was also studied, but with a more limited scope. The slope *S*_o_ is determined with an expanded uncertainty (coverage factor *k* = 2) of ± 0.008 ps/nm^2^.

Current high bit-rate telecommunication systems, both terrestrial and transoceanic, require precise information about the zero-dispersion wavelength of the installed fiber. Operating the system at a wavelength within a few nanometers of *λ*_0_ enables the use of bit rates up to and exceeding 10 Gbit/s. Knowledge of the system’s operating wavelength with respect to *λ*_0_ is also crucial for the avoidance of detrimental nonlinear effects, such as four wave mixing [4, 5].

A standard reference fiber is useful to manufacturers because of the difficulty involved in accurately measuring *λ*_0_ and *S*_0_. A National Institute of Standards and Technology sponsored interlaboratory comparison between members of the Telecommunications Industry Association (TIA), reported individual measurements of high precision, but large systematic error [6]. This is a situation where SRM calibration artifacts can be especially useful.

## 2. Frequency-Domain Phase Shift System

**Fig. 1:**
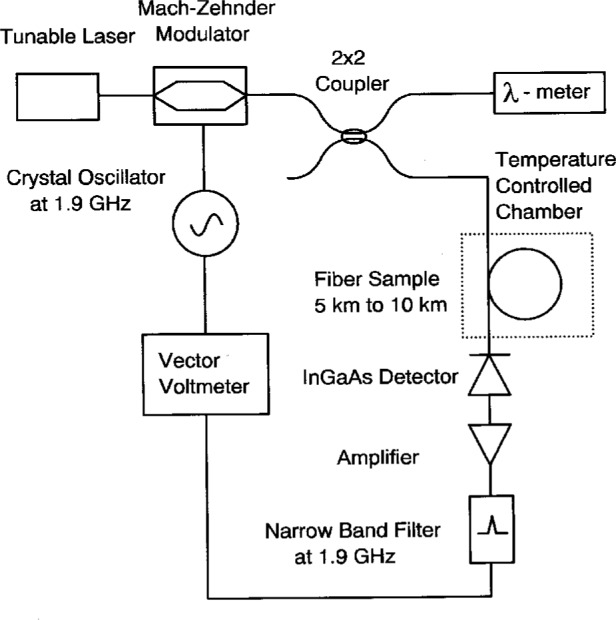
A schematic diagram of the frequency-domain phase shift system.

We have constructed a frequency-domain phase shift system designed to minimize systematic errors in measurements of *λ*_0_. Original work using this technique was performed with light-emitting diode (LED) sources [7], later systems utilizing laser diodes were developed for measuring optical fibers in both the 1.3 μm and 1.55 μm regions [8, 9]. Figure 1 shows a schematic diagram of the NIST system. Our system differs from typical implementations of the frequency-domain phase shift technique in the following ways:

1. Higher modulation frequency (1.9 GHz) to achieve a large phase angle shift per unit group delay.
2. Laser sources for increased optical power necessary to obtain sufficient signal-to-noise ratios for 0.1° phase angle resolution (electrical).
3. Interferometric monitoring of the wavelength.
4. Chirp free external modulation of the source.
5. Temperature stabilization of the fiber sample.

The continuous wave (CW) output of an external cavity tunable laser diode, with a linewidth under 2 GHz and an optical power of 1 mW, is connected to an external intensity modulator by a polarization-maintaining fiber. A grating-tuned erbium fiber laser has also been used, as an alternative source, in the 1.55 μm region. A 1.9 GHz electrical signal from a temperature-stabilized crystal oscillator is used to drive the integrated-optic Mach-Zehnder modulator. The modulated light is passed through a fiber coupler, with 20 % of the optical power being monitored by a commercial interferometric wavemeter. The remaining 80 % passes through the fiber under test. The test fiber is placed in a temperature controlled chamber whose temperature is stabilized at 23 °C (slightly above room temperature). The temperature is determined with a calibrated quartz thermometer, which has an expanded uncertainty (*k* = 2) of less than 0.1 °C. Temperature gradients within the chamber limit our fiber temperature measurement to a standard uncertainty (*k* = 1) of ± 0.15 °C. Precise control of the fiber’s temperature is necessary because the value of *λ*_0_ is temperature dependent (+ 0.030 nm/°C for dispersion-shifted fiber). The optical signal is then detected and amplified by a low noise amplifier. After the RF signal passes through a narrow bandpass filter (3 dB width of 10 MHz), it is read by the vector voltmeter (phase detector). The reference port of the vector voltmeter receives an RF signal directly from the crystal oscillator which is used as a phase reference.

Because we are interested in measuring dispersion only in the vicinity of *λ*_0_, we can use a high modulation frequency without problems due to modulo-2*p* phase uncertainty. The vector voltmeter has a phase sensitivity of 0.1°, which at our modulation frequency corresponds to a temporal resolution of 0.15 ps. The linearity of the vector voltmeter’s phase response is verified by a mechanically calibrated variable air gap which is placed in the optical beam path. After a fiber measurement, the specimen is removed, the system’s short fiber jumpers are connected and the measurement repeated; any residual dispersion in the system is subtracted out.

A dispersion measurement is performed by recording the phase from the vector voltmeter while tuning the laser over the wavelength region of interest. Each data point consists of a wavelength value and its corresponding phase. A change in group delay δ*τ*, normalized with respect to length, is related to the change in phase δ*ϕ* by the relation

$$\delta \tau =\frac{\delta \phi }{2\pi f_{m}L},$$ (1)

where *f*_m_ is the modulation frequency and *L* is the fiber length.

**Fig. 2:**
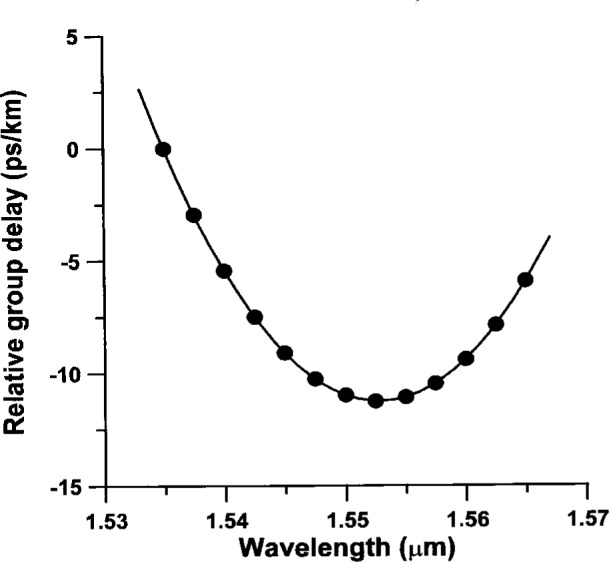
A sample fit to group delay data for a dispersion-shifted fiber.

To calculate the dispersion of a fiber specimen, we must first fit the group delay data to a theoretical curve and then differentiate the best fit group delay curve with respect to wavelength. A sample fit to group delay data is shown in Fig. 2. The dispersion coefficient *D*, defined as

$$D=\frac{d\tau }{d\lambda },$$ (2)

has the unit ps/(nm · km) and goes to zero at the so called “zero-dispersion” wavelength. Using the calculated dispersion values we can also obtain the dispersion slope (*S*),

$$S=\frac{dD}{d\lambda }.$$ (3)

The parameter *S*_o_ is defined as the dispersion slope evaluated at *λ*_0_.

For dispersion-unshifted fibers, chromatic dispersion is dominated by material dispersion. Group delay, in this case, is well described by the Sellmeier equation. Following the recommendations of Fiber Optic Test Procedure (FOTP) 169 [10], we fit the group delay data to a three-term Sellmeier equation

$$\tau (\lambda )=a\lambda^{2}+b\lambda^{-2}+c.$$ (4)

The unknown variables *a*, *b*, and *c* are solved for by the method of least squares. The group delay in a dispersion-shifted fiber has a different functional form, due to a larger waveguide contribution to dispersion. In this case, again following FOTP-169, we fit the group delay data to a quadratic equation

$$\tau (\lambda )=a\lambda^{2}+b\lambda +c.$$ (5)

There are many types of equations for fitting group delay data including: the 5-term Sellmeier, various polynomials and fits with terms involving natural logarithms. Each functional form best describes the group delay for a different class of fibers. Comparisons of these various fitting equations can be found in the literature [11, 12].

### 2.1 Determination of Group Delay

From Eq. (1) above, we can see that the uncertainty with which we can determine group delay is related to the uncertainty with which we know the modulation frequency. We measured the modulation frequency *f*_m_, which was derived from an oven stabilized crystal oscillator, to be 1920.007 MHz ± 0.002 MHz (For the rest of this paper, we will assume a coverage factor of *k* = 2 unless explicitly stated otherwise.) using a frequency counter which was calibrated in terms of NIST primary frequency standards. We measured the short term frequency stability (minutes) to be approximately a few times 10^−9^ *f*_m_. The crystal oscillator’s specified aging rate is a few times 10^−7^ *f*_m_ per month. We observed the oscillator’s output over a 7 month time span and noted shifts of no more than 3 times 10^−7^ *f*_m_. Both the short term and long term frequency behavior of the crystal oscillator are more than adequate for our uncertainty requirements. The short term frequency stability is the important parameter when measuring *λ*_0_, while the long term stability of *f*_m_ is relevant when determining *S*_0_. Absolute group delay does not need to be known to determine *λ*_0_; therefore the modulation frequency only needs to be stable for the duration of the measurement, which is a few minutes. To specify the dispersion slope *S*_0_, we need to measure absolute relative group delay.

**Fig. 3:**
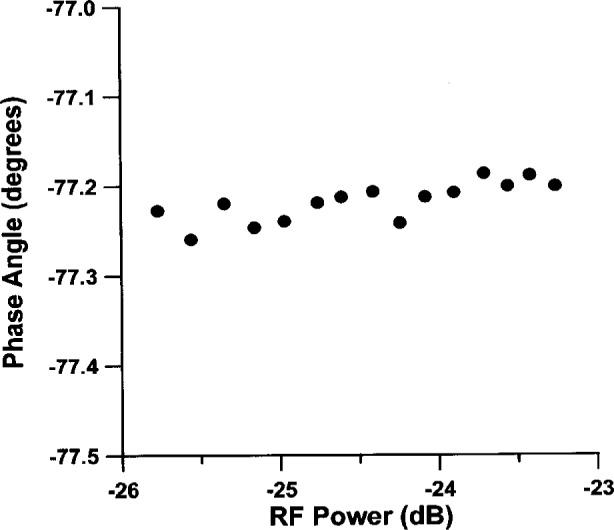
Plot of the dependence of phase upon incident RF power. The power level is in reference to 1 mW.

The vector voltmeter measures the phase angle between the RF reference signal from the crystal oscillator and the RF signal from the optical detector in the test arm. The vector voltmeter makes phase measurements which are largely independent of the input RF power level. However, the phase angle measured by the vector voltmeter still retains a weak dependence on the input power level, see Fig. 3. During a typical measurement the input power from the reference arm is held constant at level −7.6 dB below a reference level of 1 mW. The laser’s output power is not perfectly constant across the scan however; the output power varying by approximately 1 dB across the 30 nm to 40 nm wavelength scan. The variation in received RF power from the measurement arm is therefore ≈ 1 dB. These levels of power fluctuation do not have a significant effect on the phase measurements.

Two effects concerning the intensity modulation were viewed as potential sources of systematic error: the depth of modulation and drift in the modulator bias point. Both act to change the power distribution in the modulation sidebands. If either *λ*_0_ or *S*_0_ are affected by this, then there is a systematic error. The external modulator can have its bias point drift as a function of temperature; indeed, this is not uncommon with this type of integrated-optic modulator. This means that during a measurement, the modulator may not be centered on the most linear bias point, thereby introducing more power into higher order modulation sidebands. Our investigations indicated that neither the depth of modulation nor drift in the bias point caused an appreciable change in *λ*_0_ or *S*_0_.

**Fig. 4:**
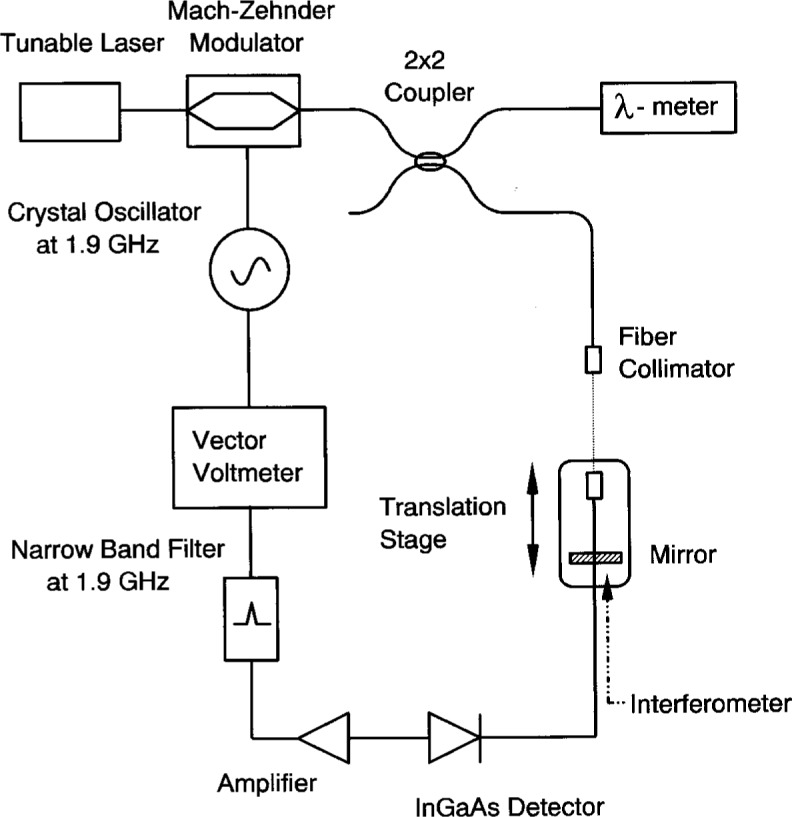
A schematic diagram of the variable air-gap used to determine the system’s linearity and accuracy in measuring group delay.

Figure 4 shows a schematic diagram of the variable air gap used to ascertain the linearity and uncertainty of the vector voltmeter’s phase response. A linear phase response is especially important for measurements of dispersion slope. The 1.9 GHz modulated optical signal is collimated in air by a lens. After traversing approximately 25 cm in air, the light was coupled back into a single-mode fiber by another collimating lens. The two collimating lenses had identical numerical apertures and are designed for optimal coupling efficiency at 1550 nm. Additionally, they are designed for low back reflection, with return losses of 45 dB. One of the collimators is mounted on a precise *X*-*Y*-*Z* translation stage. The other fiber collimator is mounted on a linear translation stage which is used to vary the length of the air gap. The stage is driven by a linear actuator with a 1 μm stepsize. To calibrate our system with respect to delay, we measure the change in the air gap distance. This is done by mounting a mirror on the back of the translation stage and measuring its position with a commercial interferometer. The details are beyond the scope of this paper, but the entire system is carefully aligned to minimize any errors due to cosine error or Abbe offset. The least count of the two frequency interferometer is less than 2 nm. The distance measured by this interferometer is traceable to a known helium-neon laser transition. The vector voltmeter was calibrated by changing the air gap over 80 mm in 2 mm steps and accurately recording its position and the corresponding RF phase measured by the vector voltmeter.

In vacuum, the modulation frequency *f*_m_ = 1.920 007 GHz has a wavelength of 0.156 141 3 m. By changing the air gap by this amount, we would expect to see a phase change on the vector voltmeter of exactly 360°. If we assume the group index for air in our laboratory is 1.000 236, the modulation wavelength in air equals 0.156 104 5 m. We experimentally measured the change in the air gap needed to induce a phase shift of 360° and compared this value to the theoretically predicted wavelength to obtain a quantitative estimate of the system’s linearity and accuracy. Phase angle (*ϕ*) as a function of air gap distance was measured many times and our values systematically differed from theory by 2 times 10^−4^*ϕ*. We attribute this difference in the phase angle to imperfections in the vector voltmeter itself. This discrepancy however, is unimportant for our purposes. The random spread in our measurements of *S*_0_ is approximately 4 times 10^−3^ *S*_0_, and so the nonlinearity in the vector voltmeter is not a limiting factor.

Laser modal noise can also contribute to phase noise. If the laser hops between different longitudinal cavity modes, each mode will propagate at a different velocity through the fiber. If the laser operates in multiple modes simultaneously, then the group arrival time will be a composite of modal arrival times. The severity of the problem depends on the length of fiber, the amount of fiber dispersion, and the magnitude of the frequency hops.

During a measurement, the system is most sensitive to modal noise when the laser is at the end of a scan, approximately 15 nm from *λ*_0_. Assuming a typical dispersion slope of 0.070 ps/(nm^2^·km), this yields a dispersion value of *D* = 10.5 ps/nm for a 10 km fiber. Since the system resolution is 0.15 ps, it would take a mode hop of 0.014 nm (1.75 Ghz at 1550 nm) to generate a large enough change in group delay to be resolvable. The tunable laser diode we are using at 1550 nm has an operating spectral width of less than 50 MHz; laser mode hops are therefore not a problem.

Optical reflections in the measurement system will cause a systematic phase error. An optical reflection will arrive out of phase with respect to the signal and add to it vectorially. We intentionally introduced optical reflections to monitor the effect on dispersion measurements. Our experimental observations agreed well with theoretical predictions. If the return losses from connectors in the system are greater than 25 dB, there should be no significant error in the measurements.

### 2.2 Determination of Wavelength

A commercial wavemeter is used to determine the vacuum wavelength of the tunable source. The wavemeter has an internal vacuum chamber and can measure *λ* with an uncertainty of 1 part in 10^6^ when the chamber is evacuated. Without evacuation there is an error due to the dispersion of the refractive index of air.

The wavemeter operates by comparing fringe counts from an internal reference laser against those produced by the unknown laser. To verify the accuracy of the wavemeter, we measured the known vacuum wavelength of a 1.523 49 μm He-Ne laser before and after every set of measurements. No systematic offset was observed between the theoretical vacuum wavelength of the He-Ne laser line and the value measured by the wavemeter [13]. All of our reported values for *λ*_0_ refer to the wavelength that would be measured in a vacuum.

### 2.3 Errors Caused by Fitting of Group Delay Data

We use the FOTP recommended fitting Eqs. (4) and (5) when fitting group delay data for dispersion-un-shifted and shifted fibers respectively. Given these fitting functions, we are interested in the dependence of *λ*_0_ on the exact manner in which the fits are implemented. Two effects were investigated: first, how *λ*_0_ changes as we change the width of the wavelength interval used in the fit; second, the sensitivity of *λ*_0_ to the centering of the group delay data about *λ*_0_. Ideally, *λ*_0_ should not depend upon either of these parameters. We investigated the dependence of *λ*_0_ on these parameters for different fiber samples: our evaluations of uncertainties are based on the largest or least favorable results observed.

**Fig. 5:**
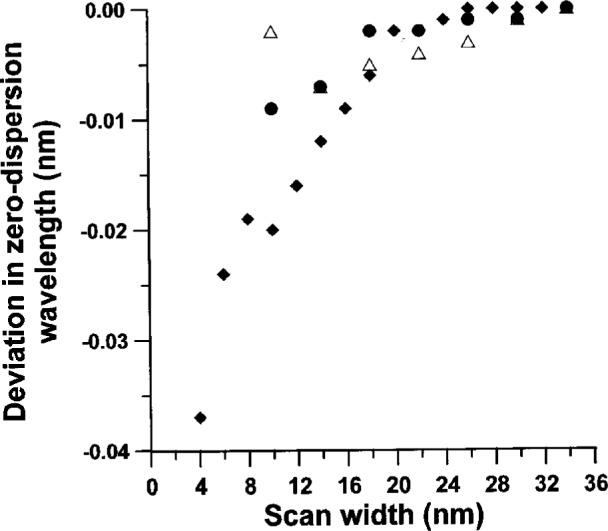
The deviation in the zero-dispersion wavelength (*λ*_0_) as a function of scan width for three fiber samples, indicated by the symbols Δ, ●, and ◆. All scans were centered about *λ*_0_ and data points were taken at 1 nm intervals.

Figure 5 shows the dependence of *λ*_0_ upon the group delay scan width. Once the wavelength range exceeds approximately 18 nm, *λ*_0_ appears to asymptotically approach a constant value. We believe smaller wavelength scans do not yield large enough changes in group delay to be immune from noise. However, fitting group delay over a very large spectral range to achieve a more global fit may well shift the value of *λ*_0_.

**Fig. 6:**
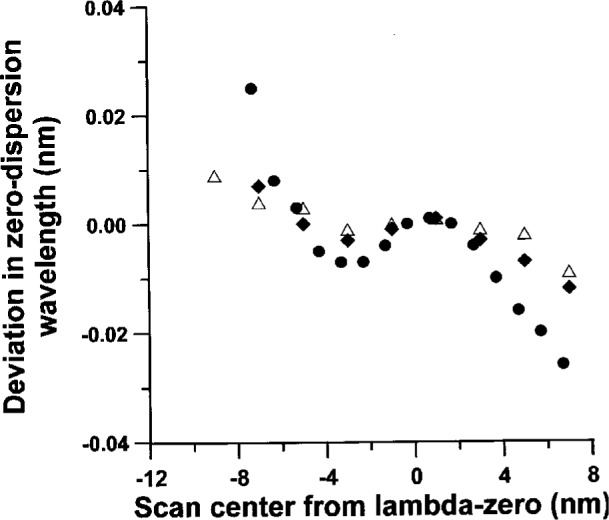
The sensitivity of the zero-dispersion wavelength (*λ*_0_) to scan centering for three fiber samples, indicated by the symbols Δ, ●, and ◆. The width of each scan was 20 nm.

How well a scan of group delay is centered about *λ*_0_ appears to have a weak effect upon the measured value of *λ*_0_. We have estimated this effect, by taking wide scans of group delay versus wavelength and then utilizing different 20 nm segments. Figure 6 shows *λ*_0_ as the symmetry is varied. Our results indicate that the general shape of the dependence seems to be repeatable from fiber to fiber, but that the magnitude of the effect varies. During an actual measurement, we estimated that scans can be centered to within 1 nm to 2 nm of *λ*_0_; therefore, we conservatively estimate the uncertainty in *λ*_0_ due to the above mentioned effects to be ± 0.007 nm (*k* = 2).

### 2.4 Effect of Chirp on *λ*_0_

Chirp is the instantaneous variation of optical frequency with time. Together with chromatic dispersion, chirp can effect how a pulse broadens and distorts as it propagates through an optical fiber [14, 15]. Depending upon whether a system is operating in the anomalous or normal dispersion region, a chirped pulse can be additionally broadened or compressed by dispersion. This makes it difficult to distinguish how much of the pulse distortion is due to chirp and how much is due to chromatic dispersion alone; ambiguous dispersion measurements can be the result.

Most directly modulated semiconductor lasers have a chirped output [16]. Changes in the injected carrier concentration result in changes in the index of refraction within the active region of the laser. The output light is shifted towards the blue or red depending upon whether the carrier concentration is temporarily above or below its equilibrium value. We avoid this problem by taking the CW output of the tunable laser and intensity modulating it with a Mach-Zehnder LiNbO_3_ integrated-optical modulator. The chirp induced by this type of modulator should essentially be zero if the propagation constants in the two arms of the modulator are equal [17].

We experimentally verified that there was no influence from residual chirp by observing the dependence of *λ*_0_ upon the modulation depth. Residual chirp could result from a difference in the mode propagation constant in each of the two interferometer arms or by a slight path difference or asymmetry between the two arms. By experimentally changing the depth of modulation we would expect to increase or decrease the contribution of chirp. No effect was observed, and we therefore assume any residual chirp in the system is negligible.

### 2.5 Uncertainty analysis

We present our uncertainty analysis in the ISO-recommended format [18]. Uncertainties were categorized as Type A, those whose distribution could be based on statistical analysis of repeated observations, or Type B, those based on scientific judgment, whose magnitude and distribution could only be estimated. Together, these uncertainties in *λ*_0_ are presented in Table 1. The type A uncertainties due to random noise and long term drift were statistically determined from fiber control charts. The expanded type A uncertainty (coverage factor *k* = 2) was estimated to be ± 0.035 nm [19]. An additional type A uncertainty due to residual dispersion in the measurement system was added in quadrature.

We estimated, assuming a normal distribution, the 2*σ* Gaussian widths for the type B uncertainties and added them in quadrature to the type A uncertainties. The uncertainty assigned to wavelength accounts for dispersion in the refractive index of air between the reference wavelength used in the wavemeter (633 nm) and the measurement wavelength (1550 nm region). The combined expanded uncertainty for measurements of *λ*_0_ was ± 0.060 nm, with a coverage factor of *k* = 2.

## 3. Differential Phase Shift System

**Fig. 7:**
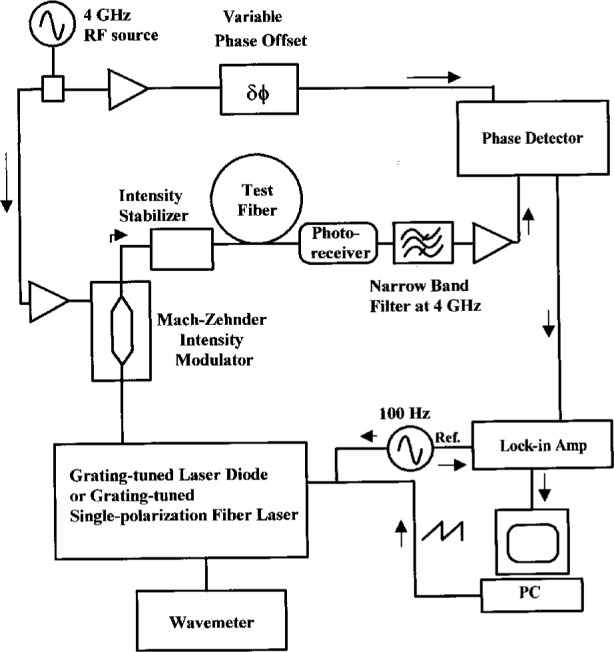
A schematic diagram of the differential phase shift system.

**Fig. 8:**
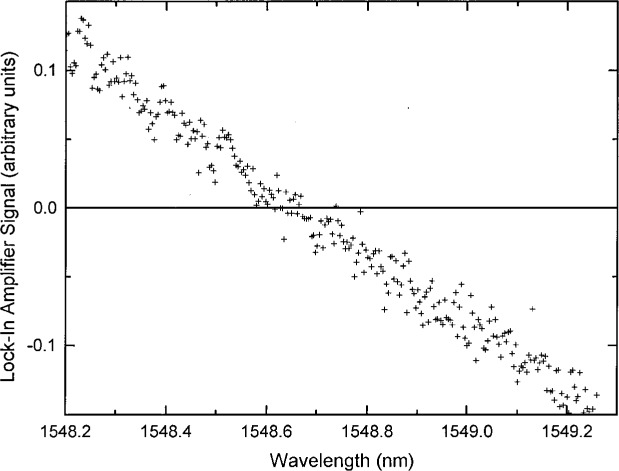
The output signal from the lock-in amplifier in the differential phase shift system as the laser is scanned over 1 nm. The above data was obtained using a 10 km fiber sample.

The second system used in our laboratory, shown in Fig. 7, is a variation of the differential phase shift technique [20, 21]. The CW output of a grating tuned laser diode or erbium-doped fiber laser (EDFL) is intensity modulated at 4 GHz. The wavelength is also dithered from 1 nm to 4 nm at a low frequency (100 Hz) by rotating a diffraction grating which is mounted on a galvanometric scanner. After passing through a low frequency intensity stabilizer, the modulated laser light is sent to the fiber under test. The optical signal is detected, filtered, and transmitted to the RF port of a quadrature mixer. The quadrature mixer operates as a phase detector and provides signals related to the phase difference between the RF and local oscillator (LO) ports. The in-phase and quadrature signals are divided to give an output proportional to tan *θ*, where *θ* is the phase angle between the RF and LO ports. By taking this ratio, the phase measurement is less sensitive to amplitude variations of the input signals. As the laser source is linearly scanned across the wavelength region of interest (typically a few nm window about *λ*_0_), the lock-in amplifier, locked to the 100 Hz dither frequency, gives an output proportional to the derivative with respect to *λ* of tan[*θ* (*λ*)]. Therefore, the output near *λ*_0_, with the proper setting of the variable phase offset, is directly proportional to the dispersion coefficient without the need for curve fitting. The entire measurement is completed in a few tens of seconds. This fast measurement time makes the system less sensitive to thermal drift of the fiber sample. This, and the elimination of curve fitting, are the principal advantages of the differential phase shift technique. At *λ*_0_, the detected output signal goes to zero; on either side of *λ*_0_, it undergoes a change in sign, as can be seen in Fig. 8.

We average multiple runs over the wavelength region about *λ*_0_. Typically, *λ*_0_ can be determined with type A expanded uncertainties approaching 0 ± 0.1 nm (*k* = 2). To obtain accurate results it is necessary to use the linear response region of the quadrature mixer, to account for residual dispersion within the system, and to ensure adequate source intensity stabilization. A measurement of the laser wavelength is made with an interferometric wavemeter (see Sec. 2.2). In our implementation of the differential phase shift technique, accurate measurements of *λ*_0_ are more technically challenging than those of the phase shift technique described in Sec. 2. In particular, the laser intensity must remain stable over the scan range and during wavelength dither. With our semiconductor laser diode, external cavity modes resulted in mode competition and mode hopping, making this difficult, especially far from the diode gain peak. With fiber laser sources, we have encountered intensity fluctuations from etalon effects in the cavity and complications due to *Q*-switching as the grating is dithered. These noise sources were minimized with external stabilization, intensity-insensitive detection, and modifications to the laser sources themselves. Nevertheless, the remaining noise from these sources placed a lower limit on the type A uncertainties obtainable with this system.

## 4. Measurement System Based on a Four-Wave Mixing Technique

We also measured *λ*_0_ using a nonlinear four-wave mixing (FWM) technique [3]. Four-wave mixing in an optical fiber is a nonlinear parametric process, where the optical fiber acts as a passive media in which the multiphoton interaction occurs [22, 23]. Conservation of energy and momentum dictate that the nonlinear process occurs efficiently only near *λ*_0_ in single-mode fibers.

**Fig. 9:**
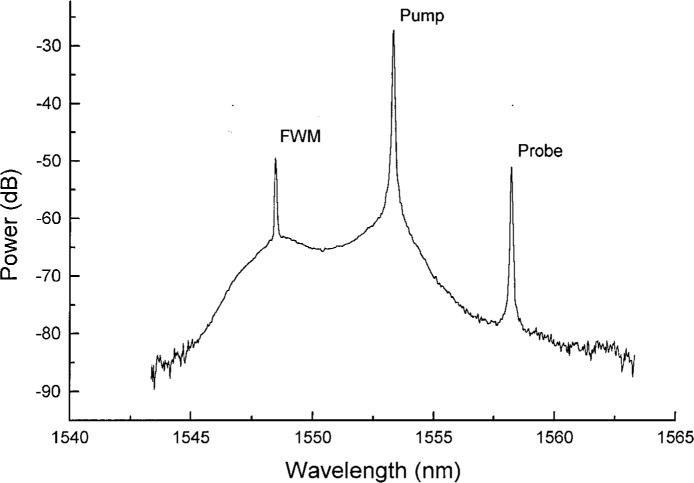
The output spectrum observed from a 10 km fiber when using the four-wave mixing (FWM) technique. When light is launched at the pump and probe wavelengths, the generated FWM signal and the probe appear symmetrically spaced with respect to the pump, which is in the vicinity of *λ*_0_. The reference power level is 1 mW.

We are interested in the “partially degenerate” case of four-wave mixing, where two of the photons have the same frequency. There are different regimes in which FWM can occur. With a strong pump laser, bound electrons are driven hard enough to elicit a nonlinear response. In this case, nonlinear effects contribute to the phase matching condition. In the case of a weak pump laser, nonlinear effects do not contribute appreciably to phase matching. The later case is the one which we utilize. In either case, the probe laser acts as a seed which stimulates generation of the FWM signal, see Fig. 9. Two pump photons combine to create two new photons, one at the probe wavelength and the other at the FWM wavelength. Energy conservation dictates that the FWM and probe signals appear symmetrically spaced about the pump wavelength. Momentum conservation dictates that the FWM process will be most efficient when the pump wavelength is at *λ*_0_.

**Fig. 10:**
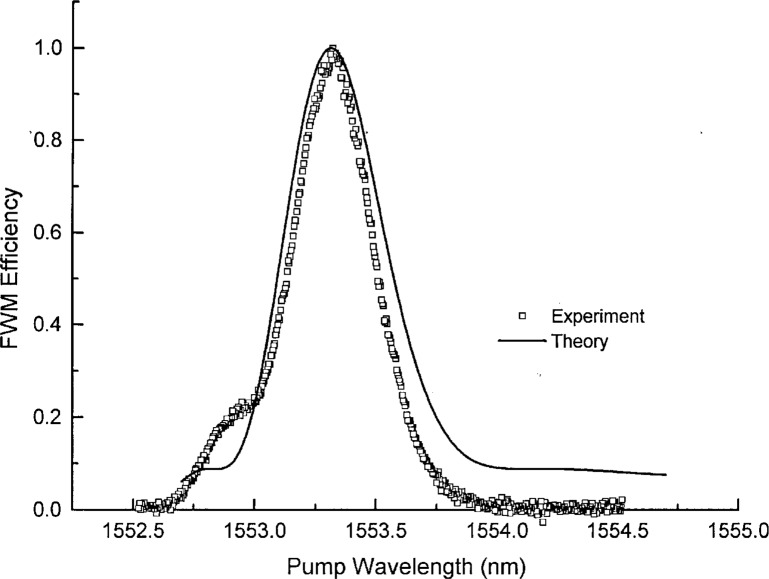
Theoretical and experimentally observed four-wave mixing (FWM) efficiency curves using a 10 km fiber with the FWM technique. At mid-scan the probe laser was 7 nm from the pump for both of these curves.

In order for momentum to be conserved, the mode propagation constants must be matched. If the phase matching condition is not precisely met, there will be a large reduction in the efficiency of the FWM process. Inoue and Toba have derived the following expression for the phase mismatch Δ*β* as a function of pump offset from *λ*_0_ [24, 25],

$$\Delta \beta =\frac{2\pi \lambda^{4}}{c^{2}}S_{0}\left(f_{1}-f_{0}\right){\left(f_{1}-f_{3}\right)}^{2},$$ (6)

where *S*_0_ is the dispersion slope at *λ*_0_, *c* is the speed of light in vacuum, *f*_1_, *f*_0_, and *f*_3_ are the frequencies of the pump, zero-dispersion wavelength, and probe respectively. It is important to note that the optimum efficiency occurs when the pump wavelength is equal to *λ*_0_. The efficiency with which a FWM signal is generated has a functional form which has the approximate shape of a sinc function (Fourier transform of a rectangle) centered about *λ*_0_. Figure 10 gives both theoretical and experimental FWM efficiency as a function of pump wavelength. In this case, the theoretical curve from Inoue [25], and the experimental data both have a mid-scan pump-probe spacing of 7 nm and a dispersion slope of 0.070 ps/(nm^2^·km). The width of the FWM efficiency peak is related to a number of parameters including the separation between the probe wavelength and *λ*_0_ and the dispersion slope at *λ*_0_ [24, 25]. The width of the FWM efficiency curve places a practical limit on the type A uncertainty with which *λ*_0_ can be determined. By tuning the pump wavelength and observing the magnitude of the FWM signal, *λ*_0_ can be identified as the wavelength where the FWM signal is produced with maximum efficiency.

**Fig. 11:**
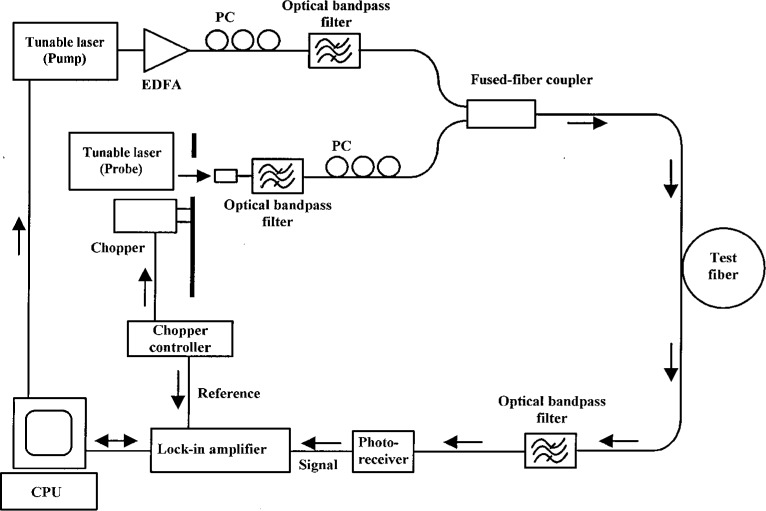
A schematic diagram of the four-wave mixing (FWM) measurement system. PC indicates a polarization controller and EDFA refers to an erbium-doped fiber amplifier.

A schematic of the experiment is illustrated in Fig. 11. The outputs of two tunable fiber lasers, one the pump and the other the probe, are passed through optical bandpass filters and polarization control paddles before being transmitted to the fiber under test. The pump laser has a CW power of +10 dB (in reference to 1 mW) after being amplified by an erbium-doped fiber amplifier (EDFA), whereas the probe laser has a CW power of −3 dB (in reference to 1 mW). The bandpass filters reduce the background amplified spontaneous emission (ASE) from the EDFA and fiber lasers. The FWM signal can be partially obscured by background ASE so it is desirable to filter the ASE to the maximum extent possible. To further reduce noise, the probe laser is mechanically chopped so the FWM signal can be synchronously detected by a lock-in amplifier at the chopping frequency. Polarization paddles are used to align the pump and probe polarization states, thereby maximizing the FWM signal.

The procedure for determining *λ*_0_ is as follows. The probe laser wavelength is selected to be within approximately 10 nm of *λ*_0_ (approximate *a priori* knowledge of *λ*_0_ is necessary). With the probe wavelength held constant, the pump laser is scanned in wavelength, and the output of the test fiber is monitored on an optical spectrum analyzer. When the pump is within a few nanometers of *λ*_0_ a FWM signal is observed. There is a range of pump wavelengths that yield an observable FWM signal, but the FWM signal is maximized when the pump wavelength is equal to *λ*_0_. During a measurement we vary the probe wavelength and look for the optimum pump wavelength for each probe setting. The measured value for *λ*_0_ should be insensitive to the probe wavelength chosen. The type A uncertainty (*k* = 2) for measuring *λ*_0_ with this system is ± 0.5 nm.

The FWM technique is sensitive to different segments of the fiber having different *λ*_0_ values. Each fiber segment will interact with the pump and probe differently to generate its own FWM signal. Therefore fibers in which *λ*_0_ varies as a function of length will have complex output spectrums, due to the superposition of different FWM signals. Meaningful information about a fiber’s *λ*_0_ value can be difficult or impossible to extract from the resulting complex patterns [26, 27]. When the fiber sample has a nominally uniform value for *λ*_0_, however, then meaningful comparisons can be made with other measurement techniques. Fibers drawn from a single preform (a large glass cylinder from which the fiber is created) should exhibit the best uniformity. We measure the maximum FWM signal (i.e., *λ*_0_) for a range of different pump polarizations; in each case polarization paddles are used on the probe laser to optimize the field overlap. In fibers with low polarization mode dispersion, polarization does not affect the value obtained for *λ*_0_. However, in other fibers, an average over polarization is taken to avoid ambiguous results.

## 5. Comparisons Between Measurement Methods

The three measurement systems discussed in this paper have been compared. We consider our frequency-domain phase shift system to be the most accurate because of its stability, low type A uncertainty, and the extensive documentation compiled for it. The differential phase shift system is not designed to measure dispersion, but rather *λ*_0_ directly. It was difficult to account for the system’s residual dispersion and other possible systematic errors (see Sec. 3). Table 2 presents comparisons between the frequency-domain and differential phase shift systems for long fibers. The average difference between the two systems is −0.07 nm, which is within the expected uncertainties of the two systems.

Table 3 presents comparisons between the FWM and the frequency-domain phase shift system. The average discrepancy between the two systems is −0.16 nm. The differences are both positive and negative, with the largest differences occurring for the shorter fibers. A number of potential systematic errors were investigated, including the effect of *λ*_0_ nonuniformity and fiber birefringence. The FWM system is a fundamentally different method for measuring *λ*_0_ in the fibers. For this reason, the agreement lends support to the uncertainty claims of the frequency-domain system.

## 6. Fiber Properties Effecting *λ*_0_

The effect of the environment is of great importance if meaningful comparisons between independent laboratories or measurement systems are to be made. Parameters whose effect on dispersion were investigated either experimentally or through the literature include: temperature, strain, and pressure. The long-term behavior of *λ*_0_ and dispersion slope were ascertained by repeated measurements over many months. Both fibers that remained in the laboratory and packaged fibers shipped around the country were periodically measured. The plotted results of these measurements were used to create “control” charts which help to monitor the long-term stability of the measurement system and the fiber samples.

### 6.1 Environmental Effects

**Fig. 12:**
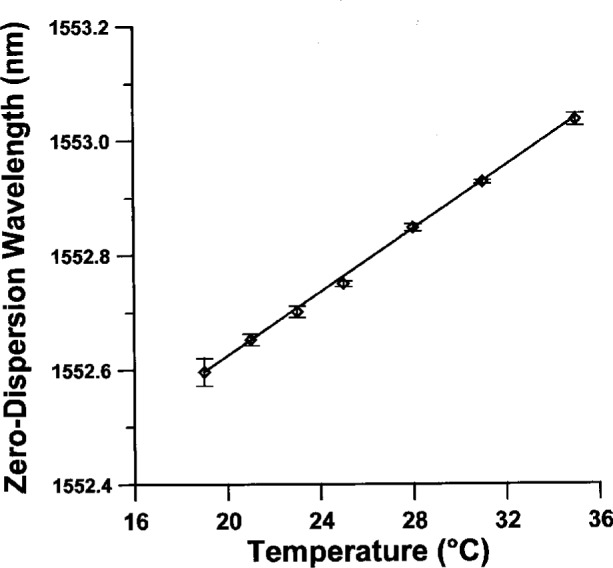
The temperature dependence of *λ*_0_ in a dispersion-shifted fiber. The slope of the line fitted to the data is + 0.028 nm/°C. Error bars are 3 times the type A standard uncertainty for each data point.

The largest environmental factor affecting *λ*_0_ is the temperature of the fiber. In dispersion-unshifted fiber samples, the temperature dependence of *λ*_0_ is + 0.025 nm/°C, while for dispersion-shifted fibers the dependence is + 0.030 nm/°C [28, 29]. There have been no reports of a temperature hysteresis effect on *λ*_0_, where the temperature was cycled over a large range (−60 °C to +250 °C) [28]. We have verified the temperature dependence of *λ*_0_ experimentally by ramping the temperature control chamber between +10 °C and +35 °C and monitoring *λ*_0_. Figure 12 shows the data and our experimentally determined temperature dependence of + 0.028 nm/°C for a dispersion-shifted fiber. The dispersion slope S_0_ has no significant temperature dependence. Before measuring fiber specimens we typically allow the fiber at least 24 hours to come to thermal equilibrium in the temperature controlled chamber.

The thermal dependence of group delay is approximately 180 ps/(°C ·km) [30]. This strong temperature dependence can lead to problems since group delay is used to measure dispersion. Changes in group delay due to temperature fluctuations can exceed the change caused by dispersion. Fortunately, thermal time constants are typically long, and if measurements of group delay are made quickly, this problem can be minimized. However, the extreme sensitivity of our system to changes in group delay makes small temperature changes significant. Data collected during a measurement may be skewed if the group delay is drifting with time (due to temperature changes). Indeed, for a 10 km fiber sample, the temperature change required to change the group delay by 0.15 ps (the resolution of our system) is only 0.1 mK.

Problems caused by thermal drift are reduced in a number of ways. First, the fiber sample is allowed to come to thermal equilibrium in a temperature controlled chamber. Second, the measurements are performed quickly, which does not allow for large temperature excursions in the sample or room. Finally, we take group delay data in two directions, first with increasing wavelength, then with decreasing wavelength. These averaged data pairs reduce errors caused by a linear drift in group delay.

We also measured *λ*_0_ in test fibers as a function of winding tension. This could be an issue, since with time the fiber may relax on the spool. We wound both dispersion-shifted and unshifted fibers at tensions varying from 0.2 N to 1.0 N (the equivalent gravitational force exerted by 20 g to 100 g masses). We observed no effect on *λ*_0_ within our measurement repeatibility. The effect of longitudinal strain upon *λ*_0_ has also been investigated by others [31]. Their findings for strain dependence, δ*λ*_0_/δ*S* = 0.015 nm/N, are consistent with our observations.

Pressure also has a small effect on *λ*_0_. Typically, this is of significance only in transoceanic undersea systems. The pressure dependence of *λ*_0_ is 0.0076 nm/MPa and can be observed in simulated transoceanic environments [31]. At oceanic depths of 8 km, extreme pressures of 82.7 MPa (12 000 psi) can be encountered. However, pressure does not have a significant effect upon SRM performance.

### 6.2 Control Chart and Longterm Stability of Fiber Samples

**Fig. 13:**
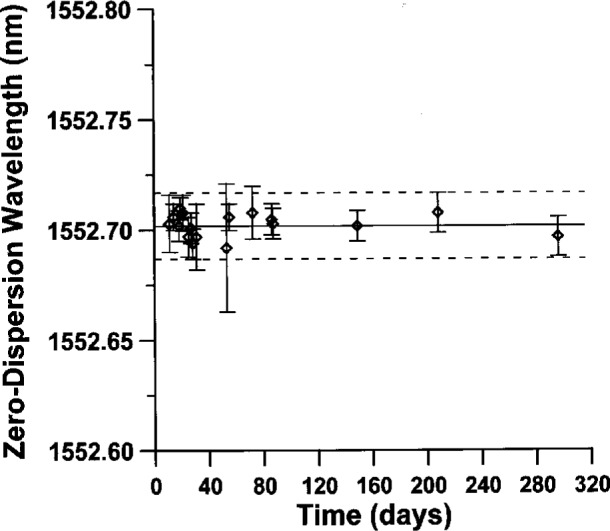
Control chart of *λ*_0_ for Fiber-F. The dashed lines represent the expanded type A uncertainty (coverage factor *k* = 3).

To determine how *λ*_0_ and the dispersion slope S_0_ are affected by fiber aging and/or relaxation, we performed repeated measurements over an 8 month time span. Figure 13 shows measured values for *λ*_0_ on a 12 km dispersion-shifted “control” fiber. We did not observe any statistically significant variations in *λ*_0_ as a function of time. Also, to determine fiber robustness, we kept control charts on fibers shipped to various laboratories for measurement intercomparisons. These specimens were exposed to vibration and large temperature fluctuations during shipping; statistically significant fluctuations in *λ*_0_ were not observed. The stability of the control fibers helps ensure the measurement system is operating correctly and no sudden systematic errors have appeared.

### 6.3 Polarization Mode Dispersion

Polarization mode dispersion (*PMD*) in single-mode fibers is caused by the breakdown of the polarization-state degeneracy in the fundamental guided mode [32]. Internal and external stresses on the fiber core and a lack of circular symmetry all lead to birefringence in the fiber and therefore to different mode propagation times for different polarizations transmitted through the fiber. The input polarizations (they need not be linear) that propagate the fastest and slowest through the fiber are known as the fast and slow principle states [33]. The difference in propagation time between the fast and slow principal states is known as differential group delay (*DGD*). *PMD* is the expected value of *DGD* within a given wavelength interval. Any other input polarization will result in a mixture between principle states with a mean arrival time somewhere in between. Since *PMD* is dependent upon the birefringence and mode-coupling properties of the optical fiber, *PMD* is time variant and best described as a statistical process. *PMD* has the unit ps/km^1/2^, where the square root dependence on length originates from the random mode coupling present in a weakly birefringent fiber (nonpolarization maintaining fiber) [34].

We have measured *PMD* values between 150 fs/√10km^1/2^ and $450\;\text{fs/}\sqrt{10\;{\text{km}}^{1/2}}$ for several packaged fiber samples. These measurements were performed with a narrow linewidth tunable laser and a commercial polarimeter using the Jones matrix eigenanalysis method [35]. The *PMD* magnitude seemed to be stable over several hours due to the stable fiber packaging and the small amount of mode-coupling. When the initially selected state of polarization was varied, using the phase shift method of Sec. 2, we could observe differences in the mode propagation time for the two principal states. The presence of first-order PMD does not directly effect dispersion measurements; however, the wavelength dependence of *PMD* or so called “second-order” *PMD* does.

Second-order *PMD* is simply the wavelength derivative of first-order *PMD* [36, 37, 38]. It has a net dispersive effect that is identical in form to chromatic dispersion. Total dispersion can be written

$$D_{\text{total}}{\left(\omega_{0}\right)}_{\pm }=D_{\text{chromatic}}(\omega_{0})\pm \frac{\Delta \tau^{\prime }(\omega_{0})}{2},$$ (7)

where Δτ*t*′ is the instantaneous wavelength derivative of first-order *PMD*,

$$\Delta \tau^{\prime }=\frac{d\Delta \tau }{d\lambda }.$$ (8)

It is important to note that as the wavelength interval over which *PMD* is averaged goes to zero, *PMD* becomes equivalent to *DGD*. In the vicinity of *λ*_0_, when chromatic dispersion approaches zero, the contribution of second-order *PMD*, can be significant and thereby cause a shift in *λ*_0_.

**Fig. 14:**
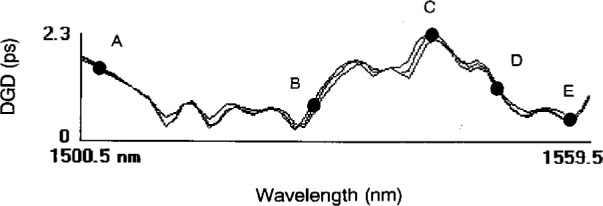
Differential group delay (DGD) as a function of wavelength for fiber artifact. Each line is a separate measurement taken hours apart. The letters indicate the wavelengths where second-order polarization mode dispersion (PMD) measurements were performed.

To verify the theory expressed by Eq. (7), we measured a high *PMD* fiber produced by winding 10 km of typical dispersion-shifted fiber onto a 1.9 cm radius spool. Winding the fiber onto a small spool induced a significant amount of bend birefringence. Additionally, the fiber winding induced mode-coupling so that the *DGD* was a function of wavelength. Figure 14 shows three measurements of the fiber’s DGD as a function of wavelength. The measurements were taken over a 2 day period. Second-order *PMD* was measured at five wavelengths, designated by the letters A through E: see Fig. 14. At these wavelengths, chromatic dispersion was measured along the principal states. Polarization control paddles were used to find the principal states and then the change in group delay over a 2 nm wavelength interval was measured for each principle state. In this manner the change in chromatic dispersion between the two principal states could be determined. According to theory, the observed difference in dispersion along the two principal states should be equal to that predicted by Eq. (7). Comparisons between the experimentally observed changes in dispersion and those predicted by theory are presented in Table 4.

The correlation between direct observations of second-order *PMD* is fairly good. One of the difficulties of the experiment is finding the principal states accurately with the polarization paddles.

**Fig. 15:**
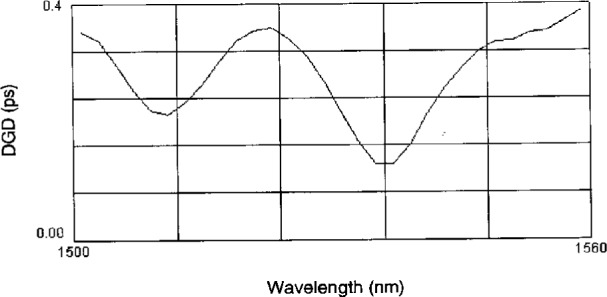
Plot of differential group delay (DGD) as a function of wavelength for fiber packaged as a Standard Reference Material.

In our packaged fiber samples, the wavelength dependence of *DGD* is quite weak; as shown in Fig. 15. We measured DGD in three 10 km fiber samples over a period of days. The largest amount of second-order *PMD* we observed was 0.011 ps/nm. If we use this value in conjunction with the typical fiber slope value (for dispersion-shifted fiber) of 0.070 ps/(nm^2^ · km), a possible shift in *λ*_0_ of 0.018 nm occurs between the two principal states. Since we will be producing many fiber SRM samples with potentially larger *DGD* values, a conservative estimate for this type B expanded uncertainty of ± 0.035 nm is adopted (coverage factor of *k* = 2).

## 7. Discussion

We have developed a frequency-domain phase shift system capable of measuring *λ*_0_, in 10 km packaged SRM fibers, with an expanded uncertainty of 0.060 nm. Most of this uncertainty originates in the measurement system itself, but we have estimated an expanded type B uncertainty in the fiber samples of ± 0.035 nm due to “second-order” polarization mode dispersion. In the near future, SRM fibers with characterized *λ*_0_ and *S*_0_ values will be commercially available from NIST [39]. Comparisons with two other measurement systems have yielded reasonable agreement.

## Figures and Tables

**Table 1:** Measurement uncertainties in *λ*_0_

	Expanded uncertainty (2*σ*) (nm)
Type A uncertainties	

Random noise (including long term stability)	0.035
Correction for residual system dispersion	0.007

Type B uncertainties	

2nd-order PMD	0.040
Curve fitting	0.007
Wavelength	0.007
Chirp	negligible
Short-term frequency stability	negligible
Temperature	0.025

Expanded uncertainty, 2*σ*	0.060

**Table 2:** Comparison of the differential phase shift and the frequency-domain phase shift techniques. Δ*λ*_0_ = *λ*_0 differential_−*λ*_0 Freq.-Domain_

Fiber	*λ*_0 Differential_ (nm)	*λ*_0 Freq.-Domain_ (nm)	Δ*λ*_0_ (nm)
C2 (10 km)	1548.7 ± 0.1	1548.86	−0.16
J (10 km)	1549.2 ± 0.1	1549.21	−0.01
F (12 km)	1552.7 ± 0.1	1552.70	0.00
J C2 (20 km)	1549.0 ± 0.1	1549.09	−0.09

**Table 3:** Comparison of the four wave mixing and frequency-domain phase shift techniques. Δ*λ*_0_ = *λ*_0 FWM_−*λ*_0 Freq.-Domain_

Fiber	*λ*_0 Differential_ (nm)	*λ*_0 Freq.-Domain_ (nm)	Δ*λ*_0_ (nm)
C2 (20 km)	1548.94	1549.17	−0.23
C2-10A (10 km)	1548.96	1548.86	+0.10
C2-10B (10 km)	1549.14	1549.44	−0.30
C2-5A (5 km)	1549.12	1549.04	+0.08
C2-5B (5 km)	1549.57	1549.87	−0.30
C2-2.5A (2.5 km)	1549.12	1548.83	+0.29
C2-2.5B (2.5 km)	1549.14	1549.22	−0.08
C2-2.5C (2.5 km)	1549.33	1549.56	−0.23
C2-2.5D (2.5 km)	1549.44	1549.94	−0.50
C2-1A (1.25 km)	1549.37	1549.82	−0.45

**Table 4:** A comparison between experiment and theory for the difference in dispersion along the two principal states of a high *PMD* fiber. Δ*D* = *D*_fast-axis_−*D*_slow-axis_

Point	Wavelength (nm)	Δ*D*, Theory (ps/nm)	Δ*D*, Experiment (ps/nm)
A	1504	0.104	0.100
B	1527	0.300	0.248
C	1541	0	0.054
D	1549	0.270	0.278
E	1557	0	0.083
